# Supplementary material for: Rotavirus infections and their genotype distribution in Rwanda before and after the introduction of rotavirus vaccination
Source: PLoS One. 2023 Apr 25;18(4):e0284934. doi: 10.1371/journal.pone.0284934 (PMC10128998; doi:10.1371/journal.pone.0284934)
Supplement: S1 Table — (DOCX) [file pone.0284934.s001.docx]

|  | Age ≤12 months | | | |  | Age 12-36 months | | | |
| --- | --- | --- | --- | --- | --- | --- | --- | --- | --- |
|  | Not vaccinated (n=330) | Vaccinated  (n=349) | OR | P value^a^ |  | Not vaccinated (n=430) | Vaccinated  (n=375) | OR | P value^a^ |
| Rotavirus (n=552) | 155 (47%) | 117 (34%) | 0.57^b^ | **0.003**^b^ |  | 128 (30%) | 133 (35%) | 0.99^b^ | 0.98^b^ |
| No Rotavirus | 175 (53%) | 232 (67%) |  |  |  | 302 (70%) | 242 (65%) |  |  |
|  |  |  |  |  |  |  |  |  |  |
| Astrovirus (rota+; n=52) | 7 (5%) | 20 (17%) | 4.36 | **0.001** |  | 3 (2%) | 22 (17%) | **8.26** | **<0.0001** |
| Astrovirus (rota–; n=54) | 7 (4%) | 10 (4%) | 1.08 | 1.00 |  | 13 (4%) | 24 (10%) | **2.45** | **0.015** |
|  |  |  |  |  |  |  |  |  |  |
| Norovirus GI (rota+; n=21) | 3 (2%) | 6 (5%) | 2.74 | 0.18 |  | 4 (3%) | 8 (6%) | 1.98 | 0.38 |
| Norovirus GI (rota–; n=29) | 6 (3%) | 11 (5%) | 1.40 | 0.62 |  | 3 (1%) | 9 (4%) | **3.85** | **0.04** |
|  |  |  |  |  |  |  |  |  |  |
| Norovirus GII (rota+; n=34) | 3 (2%) | 17 (15%) | **8.61** | **<0.0001** |  | 5 (4%) | 9 (7%) | 1.79 | 0.41 |
| Norovirus GII (rota–; n=182) | 31 (18%) | 60 (26%) | **1.62** | **0.03** |  | 35 (12%) | 56 (23%) | **2.30** | **0.0005** |
|  |  |  |  |  |  |  |  |  |  |
| Sapovirus (rota+; n=45) | 6 (4%) | 14 (12%) | **3.38** | **0.017** |  | 4 (3%) | 21 (16%) | **5.81** | **0.001** |
| Sapovirus (rota–; n=94) | 7 (4%) | 38 (16%) | **4.70** | **<0.0001** |  | 15 (5%) | 34 (14%) | **3.13** | **0.0003** |
|  |  |  |  |  |  |  |  |  |  |
| *Cryptosporidium* (rota+; n=53) | 25 (16%) | 3 (3%) | **0.14** | **0.0002** |  | 21 (16%) | 4(3%) | **0.16** | **0.0002** |
| *Cryptosporidium* (rota–; n=143) | 28 (16%) | 31 (13%) | 0.81 | 0.48 |  | 51 (17%) | 33(14%) | 0.78 | 0.34 |
|  |  |  |  |  |  |  |  |  |  |
| *Campylobacter* (rota+; n=27) | 6 (4%) | 9 (8%) | 2.07 | 0.19 |  | 5 (4%) | 7 (5%) | 1.37 | 77 |
| *Campylobacter* (rota–; n=102) | 20 (11%) | 20 (9%) | 0.73 | 0.40 |  | 36 (12%) | 26 (11%) | 0.89 | 0.67 |
|  |  |  |  |  |  |  |  |  |  |
| ETEC eltB (rota+; n=170) | 41 (26%) | 46 (39%) | **1.80** | **0.026** |  | 36 (28%) | 47 (35%) | 1.40 | 0.23 |
| ETEC eltB (rota–; n=321) | 54 (31%) | 66 (28%) | 0.89 | 0.66 |  | 108 (36%) | 93 (38%) | 1.12 | 0.53 |
|  |  |  |  |  |  |  |  |  |  |
| ETEC estA (rota+; n=108) | 30 (19%) | 28 (24%) | 1.31 | 0.37 |  | 22 (17%) | 28 (21%) | 1.28 | 0.44 |
| ETEC estA (rota–; n=183) | 35 (20%) | 44 (19%) | 0.94 | 0.80 |  | 46 (15%) | 58 (24%) | **1.75** | **0.012** |
|  |  |  |  |  |  |  |  |  |  |
| EPEC bfpA (rota+; n=84) | 23 (15%) | 28 (24%) | 1.81 | 0.06 |  | 12 (9%) | 21 (16%) | 1.81 | 0.16 |
| EPEC bfpA (rota–; n=211) | 52 (30%) | 66 (28%) | 0.94 | 0.83 |  | 38 (13%) | 55 (23%) | **2.04** | **0.002** |
|  |  |  |  |  |  |  |  |  |  |
| EPEC eae (rota+; n=128) | 35 (23%) | 32 (27%) | 1.29 | 0.40 |  | 21 (16%) | 40 (30%) | **2.19** | **0.013** |
| EPEC eae (rota–; n=269) | 55 (31%) | 69 (30%) | 0.92 | 0.75 |  | 81 (27%) | 64 (26%) | 0.98 | 1.00 |
|  |  |  |  |  |  |  |  |  |  |
| *Salmonella* (rota+; n=27) | 14 (9%) | 1 (1%) | **0.09** | **0.003** |  | 7 (5%) | 5 (4%) | 0.68 | 0.57 |
| *Salmonella* (rota–; n=64) | 12 (7%) | 14 (6%) | 0.87 | 0.84 |  | 22 (7%) | 16 (7%) | 0.90 | 0.87 |
|  |  |  |  |  |  |  |  |  |  |
| *Shigella* (rota+; n=58) | 9 (6%) | 16 (14%) | **2.57** | **0.034** |  | 10 (8%) | 23 (17%) | **2.47** | **0.025** |
| *Shigella* (rota–; n=220) | 34 (19%) | 49 (19%) | 1.11 | 0.71 |  | 79 (26%) | 58 (24%) | 0.89 | 0.62 |
|  |  |  |  |  |  |  |  |  |  |
| Astrovirus (rota+; n=52) | 7 (5%) | 20 (17%) | 4.36 | **0.001** |  | 3 (2%) | 22 (17%) | **8.26** | **<0.0001** |
| Astrovirus (rota–; n=54) | 7 (4%) | 10 (4%) | 1.08 | 1.00 |  | 13 (4%) | 24 (10%) | **2.45** | **0.015** |

Children older than 36 months were excluded because in this group only 9 were vaccinated. Data for other viruses are conditioned on absence of Rotavirus.

OR, odds ratio. ^a^ Fisher’s Exact Test.
